# Response to letter from Fontaine et al

**DOI:** 10.1016/j.jdin.2026.06.005

**Published:** 2026-06-19

**Authors:** Nupur Singh, Anna Conner, Zachary Nahmias

**Affiliations:** a Department of Dermatology, Weill Cornell Medicine, New York, New York; b Department of Psychiatry, University of South Florida, Tampa, Florida; c Department of Dermatology, NEA Baptist, Jonesboro, Arkansas

**Keywords:** biologics, delusional infestation, delusional parasitosis, delusions of parasitosis, dupilumab, Morgellons disease, neurotic excoriations, psychodermatology, treatment

*To the Editor:* We appreciate the thoughtful commentary provided by Fontaine et al which expounds upon our original article on the off-label use of dupilumab in the treatment of patients facing delusions of parasitosis (DOP).^1^

Fontaine et al showcase the potential interaction between dupilumab’s activity on interleukin (IL) 4Rα receptors in cutaneous sensory neurons and the improvement or resolution of symptoms arising from either an underlying inflammatory process or a fixed delusional belief of infestation of organisms in the body.^2^^,^^3^ Their contribution reinforces our previously published case series by again demonstrating that patients suffering from DOP often undergo multiple unsuccessful therapies ranging from antipsychotic medications and tetracycline-class antibiotics to neuromodulators and topical steroids before dupilumab is even considered.

Additional literature continues to expand this evidence base. A recent additional case report described a patient with Parkison’s disease and recalcitrant DOP who experienced complete resolution of DOP symptoms with dupilumab therapy.^4^ Combining our original case series of 11 patients with the additional 5 patients presented by Fontaine et al, and this newly reported case, a total of 17 documented patients with DOP have now shown partial or complete resolution of their symptoms following dupilumab treatment. The interpretation of these cases adds to the growing body of evidence in DOP treatment and underscores the importance of considering dupilumab in patients who have failed standard DOP therapies. Given the complexities of treating DOP, these promising outcomes may even support trialing dupilumab earlier in the treatment algorithm before initiating other options.

Given the unclear pathophysiology behind DOP, we comment on the possible involvement of the neuroimmune axis, specifically the role of IL-31 in mediating pruritic signaling in the DOP pathway. The expression of IL-31 receptor A on dorsal root ganglion neurons supports the involvement of IL-31 in neuronal itch.^5^ Consequently, we propose further investigation into nemolizumab, an IL-31 pathway inhibitor, as a possible therapeutic option in treatment of DOP. Co-administration of dupilumab and nemolizumab may also warrant exploration given a plausible synergistic effect.

We acknowledge that the experimental usage of dupilumab in treatment of this condition remains off-label and that no large-scale studies have yet clarified the exact mechanism or established statistically significant efficacy. The nature of this research is limited by small sample sizes and the absence of standardized response measures. The use of dupilumab affords dermatologists, psychiatrists, and primary care physicians a valuable emerging option for the management of this challenging condition.

## Conflicts of interest

None disclosed.
